# Supplementary material for: Monkey V1 epidural field potentials provide detailed information about stimulus location, size, shape, and color
Source: Commun Biol. 2021 Jun 7;4:690. doi: 10.1038/s42003-021-02207-w (PMC8184760; doi:10.1038/s42003-021-02207-w)
Supplement: Supplementary file 1 — Supplementary Information [file 42003_2021_2207_MOESM1_ESM.pdf]

Supplementary Figures

Monkey V1 epidural field potentials provide detailed information about stimulus location, size, shape, and color.

Benjamin Fischer and Detlef Wegener

Brain Research Institute, Center for Cognitive Science, University of Bremen, 28334 Bremen, Germany

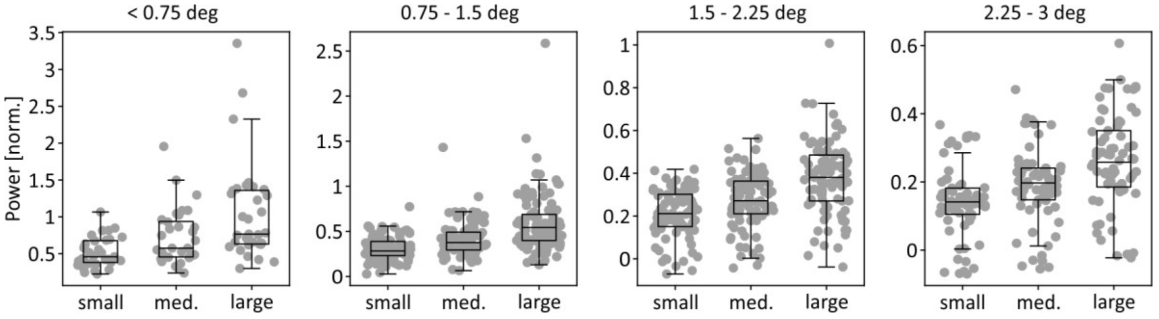

Supplementary Figure 1. Trial-averaged gamma power (y-axes) of individual channels (gray dots) as function of distance to assigned ERF (left to right) in response to small (1-degree diameter), medium (1.2-degree), and large (1.4-degree) stimuli (x-axes). Boxplot on top of individual channel data indicates median, 25th, and 75th percentile. Whiskers indicate interquartile range.

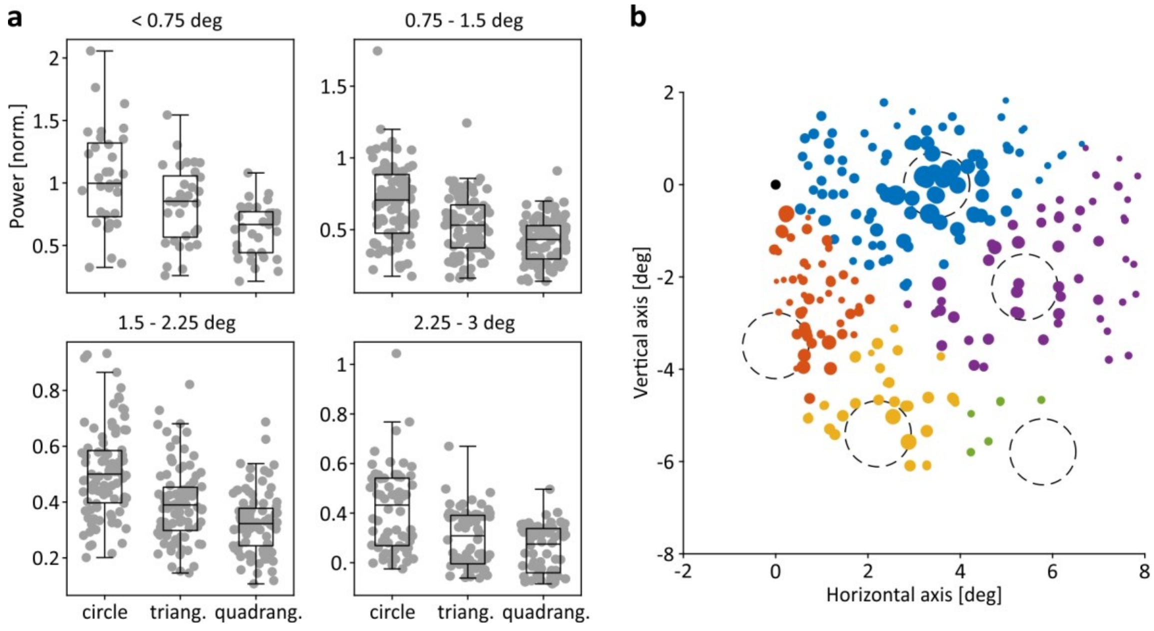

Supplementary Figure 2: (**a**) Trial-averaged gamma power (y-axes) of individual channels (gray dots) as function of distance to assigned ERF (left to right) in response to circular, triangular, and quadrangular stimuli (x-axes). Boxplot on top of individual channel data indicates median, 25th, and 75th percentile. Whiskers indicate interquartile range. (**b**) Shape sensitivity map in visual field coordinates. Colored dots represent individual EFP-channels, dot X-Y coordinates indicate center coordinates of the respective ERF, dot color represents assigned stimulus location (as indicated in Fig. 1), dot size is scaled by shape sensitivity *SSI*. Dashed circles indicate size and position of large stimuli, black dot indicates fixation point.

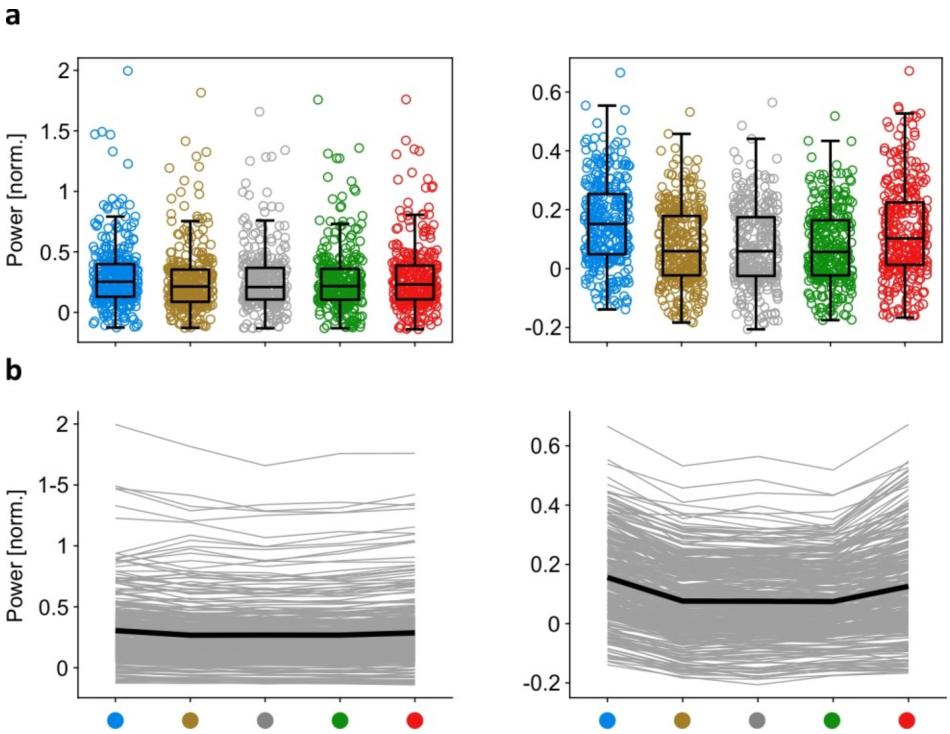

Supplementary Figure 3: (**a**) Trial-averaged gamma power (y-axes) of individual channels to each of the colors during the early (left) and late (right) response. Boxplot on top of individual channel data indicates median, 25th, and 75th percentile. Whiskers indicate interquartile range. (**b**) Paired response of single channels to each of the five colors (gray lines) during the early (left) and late (right) response. Thick black line indicates mean.
