# Supplementary material for: Monkey V1 epidural field potentials provide detailed information about stimulus location, size, shape, and color
Source: Commun Biol. 2021 Jun 7;4:690. doi: 10.1038/s42003-021-02207-w (PMC8184760; doi:10.1038/s42003-021-02207-w)
Supplement: Supplementary file 2 — Description of Supplementary Files [file 42003_2021_2207_MOESM2_ESM.pdf]

Description of Additional Supplementary Files

File name: Supplementary Data 1

Description: Excel file containing data of response matrixes in Fig.1

File Name: Supplementary Data 2

Description: Excel file containing data shown in Fig. 2

File Name: Supplementary Data 3

Description: Excel file containing data shown in Fig. 3

File Name: Supplementary Data 4

Description: Excel file containing data shown in Fig. 4

File Name: Supplementary Data 5

Description: Excel file containing data shown in Fig. 5

File Name: Supplementary Data 6

Description: Excel file containing data shown in Fig. 6

File Name: Supplementary Data 7

Description: Excel file containing data shown in Fig. 7
