# Supplementary material for: Monkey V1 epidural field potentials provide detailed information about stimulus location, size, shape, and color
Source: Commun Biol. 2021 Jun 7;4:690. doi: 10.1038/s42003-021-02207-w (PMC8184760; doi:10.1038/s42003-021-02207-w)
Supplement: Supplementary file 10 — Reporting Summary [file 42003_2021_2207_MOESM10_ESM.pdf]

nature research

Corresponding author(s): Detlef Wegener

Last updated by author(s): Apr 27, 2021

Reporting Summary

Nature Research wishes to improve the reproducibility of the work that we publish. This form provides structure for consistency and transparency in reporting. For further information on Nature Research policies, see our Editorial Policies and the Editorial Policy Checklist.

Statistics

For all statistical analyses, confirm that the following items are present in the figure legend, table legend, main text, or Methods section.

n/a	Confirmed	
☐	☒	The exact sample size (*n*) for each experimental group/condition, given as a discrete number and unit of measurement
☐	☒	A statement on whether measurements were taken from distinct samples or whether the same sample was measured repeatedly
☐	☒	The statistical test(s) used AND whether they are one- or two-sided<br>*Only common tests should be described solely by name; describe more complex techniques in the Methods section.*
☒	☐	A description of all covariates tested
☐	☒	A description of any assumptions or corrections, such as tests of normality and adjustment for multiple comparisons
☐	☒	A full description of the statistical parameters including central tendency (e.g. means) or other basic estimates (e.g. regression coefficient) AND variation (e.g. standard deviation) or associated estimates of uncertainty (e.g. confidence intervals)
☐	☒	For null hypothesis testing, the test statistic (e.g. *F*, *t*, *r*) with confidence intervals, effect sizes, degrees of freedom and *P* value noted<br>*Give P values as exact values whenever suitable.*
☒	☐	For Bayesian analysis, information on the choice of priors and Markov chain Monte Carlo settings
☒	☐	For hierarchical and complex designs, identification of the appropriate level for tests and full reporting of outcomes
☐	☒	Estimates of effect sizes (e.g. Cohen's *d*, Pearson's *r*), indicating how they were calculated

Our web collection on statistics for biologists contains articles on many of the points above.

Software and code

Policy information about availability of computer code

Data collection	Data were collected using custom-made software for visual stimulation and data acquisition. This is in-house C++ Software used for all our studies. The code can be made available upon reasonable request.
Data analysis	Data analysis was performed using custom-made Matlab code for preprocessing the data and for all subsequent analyses described in the manuscript. Detailed information on all relevant steps is provided in Methods. The code can be made available upon reasonable request. Statistical procedures were performed using Matlab's Statistics and Machine Learning Toolbox (version 11.7). Single-trial analysis was performed using the libsvm toolbox, which is open access and freely available. The toolbox is properly cited and all analyses using the toolbox are indicated in the manuscript.

For manuscripts utilizing custom algorithms or software that are central to the research but not yet described in published literature, software must be made available to editors and reviewers. We strongly encourage code deposition in a community repository (e.g. GitHub). See the Nature Research guidelines for submitting code & software for further information.

Data

Policy information about availability of data

All manuscripts must include a data availability statement. This statement should provide the following information, where applicable:

- Accession codes, unique identifiers, or web links for publicly available datasets
- A list of figures that have associated raw data
- A description of any restrictions on data availability

A data availability statement is given at the end of the manuscript, before the References.

Field-specific reporting

Please select the one below that is the best fit for your research. If you are not sure, read the appropriate sections before making your selection.

☒ Life sciences ☐ Behavioural & social sciences ☐ Ecological, evolutionary & environmental sciences

For a reference copy of the document with all sections, see nature.com/documents/nr-reporting-summary-flat.pdf

Life sciences study design

All studies must disclose on these points even when the disclosure is negative.

Sample size	Neuroscientifi data were collected from two monkeys, according to community agreement. Number of sessions per monkey was conducted to achieve about 30 trials per condition (sample size was not calculated due to unknown means and variance but conforms to experience in the field with local field potentials). Analysis was not only performed on averaged data but on single trials as well.
Data exclusions	Single trials were excluded if they contained artifacts. The trial rejection criteria and procedure is reported under Methods.
Replication	Performance of exeriments in two monkeys, with two independent epidural microelectrode arrays. All statistical findings match between animals.
Randomization	Does not apply on the level of animals. Per animal, yet, visual stimulation was fully randomized.
Blinding	Population data were analysed without blinding. Results of population data were confirmed by single-trial analysis using support vector machines (SVMs), by compiring labelled data against shuffled (blinded) data.

Reporting for specific materials, systems and methods

We require information from authors about some types of materials, experimental systems and methods used in many studies. Here, indicate whether each material, system or method listed is relevant to your study. If you are not sure if a list item applies to your research, read the appropriate section before selecting a response.

Materials & experimental systems

n/a	Involved in the study	
☒	☐	Antibodies
☒	☐	Eukaryotic cell lines
☒	☐	Palaeontology and archaeology
☐	☒	Animals and other organisms
☒	☐	Human research participants
☒	☐	Clinical data
☒	☐	Dual use research of concern

Methods

n/a	Involved in the study	
☒	☐	ChIP-seq
☒	☐	Flow cytometry
☒	☐	MRI-based neuroimaging

Animals and other organisms

Policy information about studies involving animals; ARRIVE guidelines recommended for reporting animal research

Laboratory animals	Two male macaque monkeys (macaca mulatta), 13 and 14 years old, bred for scientific purposes and obtained from the German Primate Center, Göttingen.
Wild animals	The study did not involve wild animals.
Field-collected samples	The study did not involve samples collected from the field.
Ethics oversight	The research was approved by: Die Senatorin für Gesundheit (Ministry of Health), Abteilung für Veterinärwesen (Veterinary authority), Contrescarpe 72, 28195 Bremen (based on review by the Veterinary Ethics Committee of the State of Bremen)

Note that full information on the approval of the study protocol must also be provided in the manuscript.
